# REchallenge of NIVOlumab (RENIVO) or Nivolumab-Ipilimumab in Metastatic Renal Cell Carcinoma: An Ambispective Multicenter Study

**DOI:** 10.1155/2022/3449660

**Published:** 2022-02-18

**Authors:** Charles Vauchier, Edouard Auclin, Philippe Barthélémy, Lucia Carril-Ajuria, Thomas Ryckewaert, Delphine Borchiellini, Zahra Castel-Ajgal, Mostefa Bennamoun, Luca Campedel, Antoine Thiery-Vuillemin, Elodie Coquan, Laurence Crouzet, Jean-François Berdah, Christine Chevreau, Raffaele Ratta, Aude Fléchon, Felix Lefort, Laurence Albiges, Marine Gross-Goupil, Yann-Alexandre Vano, Constance Thibault, Stéphane Oudard

**Affiliations:** ^1^Oncology Department, Hôpital Européen Georges Pompidou, AP-HP, Université de Paris, Paris 75015, France; ^2^Medical Oncology Unit, Institut de Cancérologie Strasbourg Europe, Strasbourg 67200, France; ^3^Oncology Department, Institut Gustave Roussy, Université Paris-Saclay, Villejuif 94076, France; ^4^Department of Medical Oncology, Centre Oscar Lambret, Lille 59000, France; ^5^Department of Medical Oncology, Centre Antoine-Lacassagne, Université Côte d'Azur, Nice 06100, France; ^6^Medical Oncology Department, Hôpital Cochin-Port Royal, AP-HP, Paris75014, France; ^7^Department of Medical Oncology, Institut Mutualiste Montsouris, Paris 75014, France; ^8^Department of Oncology, Hôpital Pitié Salpêtrière, AP-HP, Paris 75013, France; ^9^Department of Medical Oncology, Centre Hospitalier Universitaire de Besançon, Besançon 25000, France; ^10^Medical Oncology Department, Centre François Baclesse, Caen 14000, France; ^11^Oncology Department, Centre Eugène Marquis, Rennes 35000, France; ^12^Oncology Department, Clinique Sainte-Marguerite, Hyères 83400, France; ^13^Department of Medical Oncology, Institut Universitaire du Cancer Toulouse-Oncopole, Toulouse 31100, France; ^14^Oncology and Supportive Care Department, Hôpital Foch, Suresnes 92150, France; ^15^Department of Medical Oncology, Centre Léon Bérard, Lyon 69008, France; ^16^Department of Medical Oncology, Centre Hospitalier Universitaire de Bordeaux-Hôpital Saint-André, Bordeaux 33000, France; ^17^Centre de Recherche des Cordeliers, INSERM, Université de Paris, Sorbonne Université, Paris 75006, France; ^18^Université de Paris, PARCC, INSERM U970, Paris 75006, France

## Abstract

**Introduction:**

Immune checkpoint inhibitors (ICI) have been approved for front-line therapy in metastatic renal cell carcinoma (mRCC). However, progressive disease often occurs and subsequent therapies are needed. ICI rechallenge may be an option, but there is a lack of data regarding efficacy and prognostic factors. We assessed efficacy of ICI rechallenge and factors associated with better outcomes. *Patients and Methods*. This ambispective multicenter study included 45 mRCC patients rechallenged with nivolumab ± ipilimumab between 2014 and 2020. Primary endpoint was investigator-assessed best objective response rate (ORR) for ICI rechallenge (ICI-2). Factors associated with ICI-2 progression-free survival (PFS) were evaluated with multivariate Cox models.

**Results:**

ORR was 51% (*n* = 23) at first ICI therapy (ICI-1) and 16% (*n* = 7) for ICI-2. Median PFS was 11.4 months (95% CI, 9.8–23.5) and 3.5 months (95% CI, 2.8–9.7), and median overall survival was not reached (NR) (95% CI, 37.8–NR) and 24 months (95% CI, 9.9–NR) for ICI-1 and ICI-2, respectively. Factors associated with poorer ICI-2 PFS were a high number of metastatic sites, presence of liver metastases, use of an intervening treatment between ICI regimens, Eastern Cooperative Oncology Group performance status ≥2, and poor International Metastatic RCC Database Consortium score at ICI-2 start. Conversely, ICI-1 PFS >6 months was associated with better ICI-2 PFS. In multivariate analysis, there were only statistical trends toward better ICI-2 PFS in patients with ICI-1 PFS >6 months (*p*=0.07) and toward poorer ICI-2 PFS in patients who received a treatment between ICI regimens (*p*=0.07).

**Conclusion:**

Rechallenge with nivolumab-based ICI has some efficacy in mRCC. We identified various prognostic factors in univariate analysis but only statistical trends in multivariate analysis. Our findings bring new evidence on ICI rechallenge and preliminary but unique data that may help clinicians to select patients who will benefit from this strategy.

## 1. Introduction

Sunitinib and pazopanib, two antiangiogenic tyrosine kinase inhibitors (TKI), were considered as the first-line standard of care for metastatic RCC (mRCC) for a long period of time, regardless of the International Metastatic RCC Database Consortium (IMDC) prognostic score [1, 2]. Subsequently, immune checkpoint inhibitors (ICI) have been approved in mRCC treatment. Firstly, in previously treated mRCC, nivolumab, an anti-programmed death-1 (PD-1) antibody, showed overall survival improvement compared to everolimus [3]. Then, for front-line therapy, immune-base combination treatments were validated. Nivolumab-ipilimumab (an anti-cytotoxic T-cell lymphocyte antigen-4 antibody (CTLA-4)) combination improved overall survival (OS) for intermediate and poor IMDC prognostic score of clear cell RCC patients [4]. Different TKI-ICI combinations as axitinib-pembrolizumab (an anti-PD-1 therapy), axitinib-avelumab (an anti-programmed death-ligand-1 (PD-L1) antibody), or, more recently, cabozantinib-nivolumab and lenvatinib-pembrolizumab also improved outcomes in first-line treatment [5–8].

Hence, immune-based combinations became new standard for previously untreated mRCC. However, treatment discontinuation often occurs. Subsequent treatments include different antiangiogenic compounds. The question whether the rechallenge of ICI may be an option in mRCC remains unanswered. This strategy seems to be reasonably safe [9–11] and has shown some efficacy according to nonrandomized studies in metastatic melanoma [12–14] or in non-small cell lung cancer (NSCLC) [15–17]. In mRCC, few retrospective and nonrandomized prospective studies showed a modest efficacy of ICI rechallenge [18–21]. However, most of them focused only on ICIs combination rechallenge and no prognostic factors of response were assessed.

Therefore, we collected data from a national cohort of mRCC patients who underwent ICI rechallenge with nivolumab or nivolumab-ipilimumab combination in order to explore efficacy, safety, and potential prognostic factors associated with better outcomes in this strategy.

## 2. Patients and Methods

### 2.1. Study Design

This ambispective observational multicenter study included mRCC patients from 16 French centers. We provided to the patients written information about the study's objectives and the nature of the information that we collected. Given the noninterventional nature of the study, ethic committee approval was not required. The study was registered in the public repertory of the French health data institute (Institut National des Données de Santé) and conformed to the MR no. 004 form of the French data protection agency (Commission Nationale de l'Informatique et des Libertés).

### 2.2. Patients

We included patients with mRCC of all histological subtypes who received a rechallenge with nivolumab ± ipilimumab between January 2014 and September 2020. ICI rechallenge was defined as the second regimen of ICI (ICI-2) administered after a prior ICI therapy (ICI-1). Between the two ICI regimens, patients could have either a prolonged treatment-free interval (≥12 weeks) or a treatment period with at least one TKI. ICI discontinuation could occur after a disease progression (PD), an ICI-related toxicity, or a clinical decision. A clinical decision for ICI discontinuation was defined as the physician's choice based on a long-lasting nonprogressive disease under ICI therapy, without limiting toxicity and patient's wish for a pause in treatment. Patients who resumed ICI-2 therapy without prior disease progression were excluded. Treatment's efficacy was assessed by clinical evaluations and CT scans on a regular basis. Radiologic assessments were locally performed using the Response Evaluation Criteria in Solid Tumor version 1.1 (RECIST v1.1). Patients without a follow-up scan after beginning ICI rechallenge were considered nonevaluable. Immune-related adverse events (irAEs) type and grade were reported according to the Common Terminology Criteria for Adverse Events version 5.0 (CTCAE v5.0). All patients were treated according to their own's physician decisions.

### 2.3. Data Collection

We collected the following data from patients' medical files: demographic data including gender, age at diagnosis, tumor characteristics as histological type, Fuhrman and/or International Society of Urological Pathology (ISUP) nuclear grade, and presence of sarcomatoid features; and data for each regimen as treatment administered, rank of line, Eastern Cooperative Oncology Group (ECOG) performance status (PS), metastatic sites, IMDC score, blood test results at baseline, date of first and last cycles, date of first occurrence of a response, best achieved response, type and grade of irAE, and reason for treatment discontinuation.

### 2.4. Endpoints

The primary endpoint of the study was the objective response rate (ORR) for ICI-2. Secondary endpoints were duration of treatment (DOT), duration of response (DOR), progression-free survival (PFS), OS, safety for ICI-1 and ICI-2, and prognostic factors for ICI-2 PFS. All the endpoints were locally assessed for both ICI regimens.

### 2.5. Statistical Analyses

Median values (interquartile range) and frequencies (percentage) were provided for description of continuous and categorical variables, respectively. Medians and proportions were compared using Student's *t*-test and chi-square test (or Fisher's exact test, if appropriate), respectively.

DOT was defined as the time between the first administration and the last administration of therapy. DOR was defined as the time between first occurrence of response and progression or death of any cause. PFS was defined as the time between start of therapy and disease progression or death of any cause, whichever occurred first. OS was defined as the time between start of therapy and death of any cause.

PFS and OS were estimated using the Kaplan-Meier method and described using median or rate at specific time points with their 95% confidence intervals (95% CIs). Follow-up was calculated using the reverse Kaplan–Meier method. Patients who were still alive and undergoing treatment at final analysis were censored at the date of last visit. The association between clinical and biological factors and ICI-2 PFS was assessed with univariate and multivariate Cox models.

ORR was defined as the percentage of patients who achieved complete response (CR) or partial response (PR) as best response and disease control rate (DCR) as the percentage of patients who had a nonprogressive disease as best response.

Statistical analyses were performed on R v.4.0.3. All *p* values <0.05 were considered statistically significant.

## 3. Results

### 3.1. Population

We included a total of 45 patients with nivolumab ± ipilimumab rechallenge. Median duration of follow-up was 14.9 months (95% CI, 12.0–20.7). 64% of the patients were males and median age was 59 years (range: 42–90). Most of them had clear cell histology (91%) and a Fuhrman or ISUP nuclear grade ≥3 (79%) and 12% had associated sarcomatoid tumor. Thirty-nine (87%) patients underwent partial or radical nephrectomy. For all of them, it occurred just after the diagnosis of RCC, at a localized stage for 31 (80%) patients, and at metastatic stage for 8 (21%) patients (Table 1).

Compared to ICI-1, at ICI-2, patients had poorer ECOG PS (PS ≥ 2 in 23% at ICI-2 versus 2% at ICI-1), greater metastatic disease burden (≥3 sites of disease in 56% at ICI-2 versus 34% at ICI-1) and poorer IMDC score (poor risk in 53% at ICI-2 versus 12% at ICI-1). During ICI-2, 93% (*n* = 42) received nivolumab alone and 7% (*n* = 3) nivolumab-ipilimumab combination. During ICI-1, 78% (*n* = 35) received nivolumab and 11% (*n* = 5) nivolumab-ipilimumab, 4% (*n* = 2) received an anti-PD-L1 monotherapy, and 7% (*n* = 3) received a TKI-ICI combination (avelumab-axitinib, nivolumab-tivozanib, and pembrolizumab-lenvatinib). Twelve patients participated in clinical trials during their first ICI regimen (studies and ClinicalTrial.gov numbers: Checkmate025, NCT01668784 [3]; NIVOREN, NCT03013335; IMmotion150 NCT01984242; JAVELIN Solid Tumor, NCT01772004; JAVELIN Renal 101, NCT02684006 [6]; CLEAR, NCT02811861 [8]; Checkmate 214, NCT02231749 [4]; TiNivo, NCT03136627; and BIONIKK, NCT029609). Median numbers of prior lines of therapy were 3 (range, 1–9) and 1 (range, 0–6), respectively, for ICI-2 and ICI-1. ICI-1 discontinuation occurred after PD in 49% (*n* = 22) patients, ICI-related toxicity in 27% (*n* = 12), and clinical decision in 24% (*n* = 11). Details of clinical decisions leading to ICI-1 discontinuation are listed in Supplementary Table S1. Between ICI regimens, 42% (*n* = 19) patients had a prolonged treatment-free interval and 58% (*n* = 26) received at least one TKI regimen (Table 2, Supplementary Figure S1). They all experienced PD before starting the therapy subsequent to ICI-1.

### 3.2. Efficacy of ICI-1 Regimen

ORR at ICI-1 was 51% (Supplementary Table S2). Four (9%) patients achieved CR and 19 (42%) had PR as best response. DCR was 76%, median DOT was 5.7 months (95% CI, 3.5–12.2), median PFS was 11.4 months (95% CI, 9.8–23.5), and median OS was not reached (NR) (95% CI, 37.8–NR).

### 3.3. Efficacy of ICI-2 Regimen

Primary endpoint of ORR at ICI-2 was 16%. DCR at ICI-2 was 47%. Median DOT was 3.5 months (95% CI, 2.5–9.8). For patients who responded, median DOR was 5.1 months (95% CI, 2.7–NR). Median PFS was 3.5 months (95% CI, 2.8–9.7) and median OS was 24 months (95% CI, 9.9–NR).

### 3.4. Description of Responders

Under ICI-2, one (2%) patient achieved CR and six (13%) patients had PR (Table 3). They all received nivolumab alone at rechallenge. The only patient achieving CR at ICI-2 had also achieved CR at ICI-1 under nivolumab-ipilimumab combination. ICI-1 was discontinued for disease progression and the patient received one TKI regimen before resuming ICI-2. Three patients achieved PR at ICI-1. One was treated with nivolumab-ipilimumab at ICI-1 that was discontinued for toxicity. He resumed ICI-2 therapy after a 9-month-long treatment-free interval. The other two patients received nivolumab at ICI-1 which was discontinued for clinical decision. They also had a treatment-free interval of 5 and 12 months before starting ICI-2. One patient experienced PR at ICI-2 after achieving stable disease (SD) under nivolumab monotherapy at ICI-1. Finally, two patients experienced PR at ICI-2 after PD as best response at ICI-1. One of them discontinued nivolumab for progression and resumed ICI-2 after two lines of TKI. The other underwent three regimens of nivolumab in his disease course. Each rechallenge was considered independently and compared to the first ICI therapy he received. PD occurred, as best response, at first and second nivolumab regimen but PR was achieved at second rechallenge. He received one TKI regimen between each ICI round. Disease progression eventually occurred after 8 months of ICI rechallenge.

### 3.5. Factors Associated with ICI-2 Benefit

#### 3.5.1. Objective Response and Disease Control

**Figure 1:**
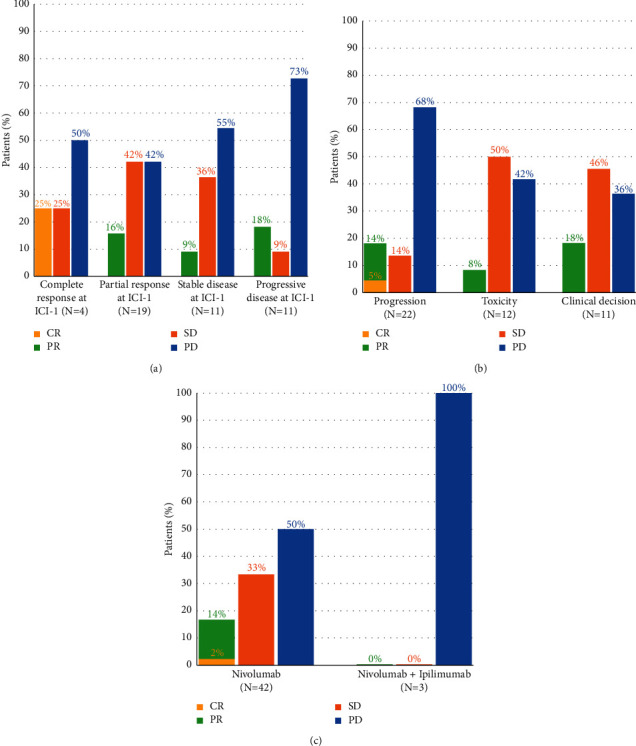
Response to ICI-2 stratified by (a) best response to ICI-1 (*p*=0.31), (b) reason for ICI-1 discontinuation (*p*=0.18), and (c) therapy received at ICI-2 (*p*=0.57).CR, complete response; PR, partial response; SD, stable disease; PD, progressive disease.

Response to ICI-2 did not significantly differ after stratification on the response to ICI-1 (*p*=0.31), the reason for ICI-1 discontinuation (*p* = 0.18), or the treatment received at rechallenge (*p*=0.57) (Figure 1). No difference was seen in ICI-2 ORR regarding the response to ICI-1 (*n* = 5/23 (17%) for patients with CR/PR at ICI-1; *n* = 2/11 (18%) for patients with PD at ICI-1) or the reason for ICI-1 discontinuation (toxicity: *n* = 1/12 (8%); clinical decision: *n* = 2/11 (18%); PD: *n* = 4/22 (18%)). However, DCR was higher in patients who achieved CR (*n* = 2/4 (50%)), PR (*n* = 11/19 (58%)), and SD (*n* = 5/11 (45%)) as best response to ICI-1 than in those who had PD (*n* = 3/11 (27%)). DCR was also higher in patients who discontinued ICI-1 for toxicity (*n* = 7/12 (58%)) or clinical decision (*n* = 7/11 (64%)) compared with those who discontinued ICI-1 for progression (*n* = 7/22 (32%)).

#### 3.5.2. Progression-free Survival

**Figure 2:**
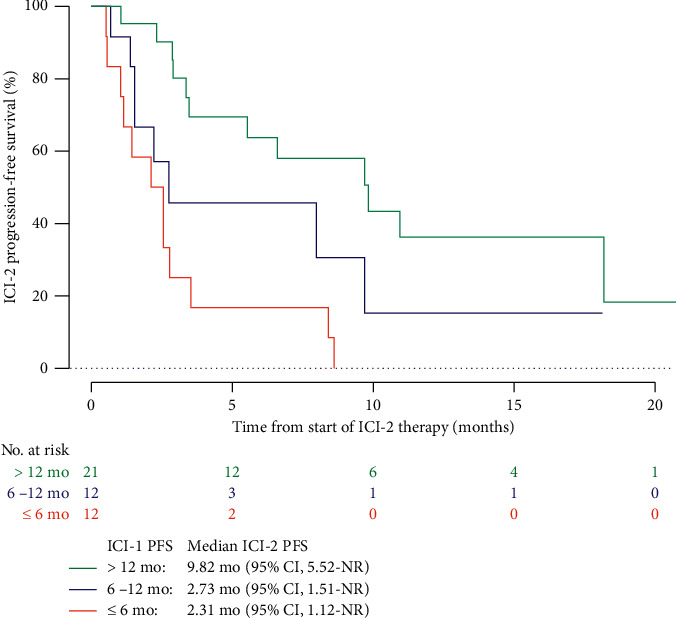
ICI-2 progression-free survival according to ICI-1 PFS. PFS, progression-free survival; CI, confidence interval; NR, not reached; mo, months.

In univariate analysis (Supplementary Figure S2), characteristics associated with a poor ICI-2 PFS were as follows: ECOG PS ≥ 2 at ICI-2 start, big number of metastatic sites, presence of liver metastases, use of an intervening treatment between ICI regimens, and a poor IMDC score at ICI-2 start. ICI-1 PFS >6 months was associated with better ICI-2 PFS (Figure 2). Clinicopathological features as histological subtype, nuclear grade, or sarcomatoid features were not significantly associated with ICI-2 PFS, as well as nephrectomy status.

**Figure 3:**
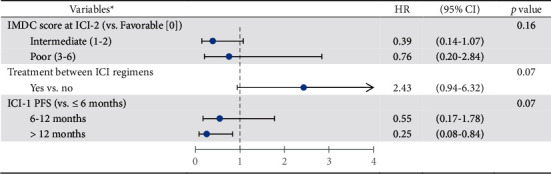
Factors associated with PFS at ICI-2 (multivariate analysis). *HR, hazard ratio; CI, confidence interval; IMDC, International Metastatic Renal Cell Carcinoma Database Consortium; ICI, immune checkpoint inhibitors; PFS, progression-free surviva*l. *Note*. ^*∗*^Among significant factors in univariate analysis, the three most relevant variables were selected for multivariate analysis. The others were not included due to a lack of power.

In our multivariate analysis (Figure 3), after adjustment on IMDC score and the use of a intervening treatment between ICI regimens, none of the variables were significantly associated with a better PFS at ICI-2. There was a trend toward better ICI-2 PFS in patients with prolonged ICI-1 PFS (6 to 12 months versus ≤ 6 months: hazard ratio (HR) 0.55, 95% CI 0.17–1.78; >12 months versus ≤ 6 months: HR 0.25, 95% CI 0.08–0.84; *p*=0.07). Conversely, a trend toward poorer ICI-2 PFS was observed in patients receiving a treatment between ICI regimens (HR 2.43, 95% CI 0.94–6.32; *p*=0.07).

### 3.6. Safety of ICI Rechallenge

During ICI-1, 29% (*n* = 13) patients experienced limiting or serious irAE. Twelve of them led to ICI-1 discontinuation. During ICI-2, 4% (*n* = 2) patients had an irAE. One experienced a recurrence of his first irAE (psoriasis) with the same severity (grade 3), and one had a new irAE (nephritis grade 3). Hence, risk of recurrence of the initial irAE was 8% (*n* = 1/13) and there was no difference in severity (Supplementary Table S3). Among data available, 10% (*n* = 4) patients received systemic corticosteroid during ICI-2. No immune-related death was reported.

## 4. Discussion

In our cohort of 45 patients with mRCC rechallenged with an ICI, we showed some efficacy with a 16% objective response rate and a favorable safety profile. The CHECKMATE 025 study [3], comparing nivolumab versus everolimus in previously treated ICI-naive mRCC, showed an ORR of 25%, a median PFS of 4.6 months, and a median OS of 25 months. In this study, nivolumab was the first ICI regimen that patients received and only 28% were treated at third line or more. Although our cohort is different with more advanced diseases and patients previously treated with ICI, our findings suggest that nivolumab or nivolumab-ipilimumab seem to keep some activity in a rechallenge situation.

Retrospective studies by Ravi et al. [18] and Gul et al. [19] reported comparable ORR of 23% and 20%, respectively, for ICI rechallenge in mRCC. In Ravi et al.'s study, 69 patients were rechallenged with ICI: 38% received ICI in monotherapy, 32% in combination with other ICIs, and 19% in combination with TKIs. Median PFS was 5.7 months. In Gul et al.'s study, 45 mRCC patients were rechallenged with nivolumab-ipilimumab with a median DOR of 7 months. In this study, median PFS was 3.5 months and median DOR was 5.1 months at rechallenge, which seem less promising. This may be explained by a more advanced disease course in our population with a poorer ECOG PS (PS ≥ 2: 23% in ours versus 7% in Gul et al.'s study) and a higher median of prior systemic therapy (3 [range, 1–9] in ours versus 2 [range, 1–7] in Ravi et al.'s study and 3 [range, 1–7] in Gul et al.'s study). Moreover, in our study, nivolumab was mostly administered alone which may influence the outcome. Similar results were found in retrospective studies that assessed ICI rechallenge in NSCLC. Gobbini et al. [15] collected data of 144 NSCLC patients, rechallenged mostly with anti-PD-1 monotherapy (94%) at third or later line (79%). 18% were ECOG PS ≥ 2 at start of rechallenge. ORR was 16% and median PFS was 4.4 months. In Levra et al.'s study [16], most of the 1517 NSCLC patients received nivolumab at rechallenge with a median DOT up to 4.0 months.

It is also of note that, in those real-world setting retrospective studies of ICI rechallenged mRCC, outcomes at first ICI seem rather substantial compared to historic data [3–7], showing that patients undergoing a rechallenge are already selected patients. Four ongoing nonrandomized prospective phase II trials explored salvage nivolumab-ipilimumab after prior ICI in mRCC: HCRN GU16-260 (Atkins et al., ASCO 2020 (Abstract)), FRACTION (Choueiri et al., ASCO 2020 (Abstract)), TITAN (Grimm et al., ESMO 2019 (Abstract)), and OMNIVORE [20] studies. Their results were pooled in a recent meta-analysis [21]. Those studies reported an ORR of 4% to 15% during nivolumab-ipilimumab salvage therapy, with a pooled ORR of 10% in the meta-analysis. The FRACTION study reported a median DOR of 13.8 months and a median PFS of 7.4 months during salvage therapy. Studies had different design and heterogeneity in their population. The FRACTION study included only patients with progressive disease under prior ICI, whereas the other three included nonresponder patients. Patients in the OMNIVORE study also received fewer cycles of salvage ipilimumab.

Our study is the first to our knowledge that assessed prognostic factors for benefit of ICI resumption. Ravi et al.'s study showed that patients with a response at first ICI therapy were more likely to respond at rechallenge, which was not seen in our study. Nonetheless, no prognostic factors were assessed [18], as in Giaj Levra et al.'s study [16]. Gobbini et al. [15] reported in NSCLC patients that absence of chemotherapy between ICI regimens, discontinuation of first ICI due to toxicity or clinical decision, and low number of metastatic sites were associated with better PFS at rechallenge in univariate analysis. Only ECOG PS was significant in multivariate analysis. Concerning metastatic melanoma, good ECOG PS and low lactate dehydrogenase count were associated with longer OS under nivolumab-ipilimumab combination after prior anti-PD-1 therapy in univariate analysis [12].

The discussion of a rechallenging strategy should consider patients' characteristics and first response to ICI. In univariate analysis, ICI-1 PFS >6 months positively affected ICI-2 PFS. Conversely, factors associated with poorer ICI-2 PFS were ECOG PS ≥ 2, a big number of metastases, presence of liver metastases, treatment used between ICI therapies, and intermediate or poor IMDC score. These factors reflect a more aggressive and a more advanced disease. A high tumor burden with big number of metastatic sites also affected ICI-2 PFS, although the nephrectomy status did not. The comparison between immediate and delayed (from diagnosis) nephrectomy might be of interest in this population but it could not be assessed in our study since nephrectomy occurred at diagnosis for all concerned patients. ICI-2 PFS was not influenced by histological subtypes, nuclear grade, or presence of sarcomatoid features. This could be explained by a few number of patients in those subgroups: only four patients had a different histology (one papillary, one chromophobe, and two TFE3-translocation RCCs), eight had a low nuclear grade, and five had sarcomatoid features. In multivariate analysis, no variable was statistically associated with the PFS at ICI-2. Receiving a systemic treatment between ICI regimens tended to be associated with a shorter PFS, while ICI-1 PFS >6 months tended to be associated with a prolonged PFS at rechallenge. There was only a trend toward a statistical significance, probably owing to a lack of power for multivariate analysis. However, we may assume that patients with good disease control under first ICI regimen, characterized by an ICI-1 PFS >6 months and no need for a treatment between ICI regimens, are more likely to have a prolonged PFS at rechallenge. Larger prospective studies are needed to confirm these results.

Interpretation of our findings needs to take into account the reason for ICI-1 discontinuation, the use of TKI between ICIs, the number of previous treatments, and type of ICI used since it might influence the outcome.

Patients who discontinued ICI-1 for PD tend to have a poorer ICI-2 PFS compared to those who discontinued it for toxicity or clinical decision and thus did not experience disease progression under ICI-1. This difference may be due to a more aggressive disease, a less ICI-responsive tumor, or the acquisition of a resistance to ICI in the PD subgroup [22]. Therefore, those two groups may represent two distinct situations regarding the response to a rechallenge of ICI. In our study, we decided to include both groups for the analyses in order to assess efficacy and prognostic factors in a more general population and limit the loss of power for statistical analyses. Thus, larger-cohort studies are necessary to explore the outcomes in each of these groups.

Besides, patients who experienced PD under ICI-1 mostly received one or more TKI regimens before ICI-2 readministration, whereas patients who discontinued ICI-1 for toxicity or clinical decision were more likely to immediately resume ICI when PD occurred. Switching treatment for an antiangiogenic TKI is a common practice regarding this situation of progressive disease, since few data support the strategy of rechallenging ICI and that there is a need for a better disease control. However, receiving a TKI treatment between the two ICI rounds might influence PFS at rechallenge, eventhough it is not significant in our multivariate analysis.

RCC is a highly vascularized and immunogenic tumor type with great efficacy of ICI-based combinations. However, a significant number of patients (22% of patients in Checkmate 214 study [4]) have a progressive disease as best response, suggesting a primary resistance to ICI. Prolonged responses under ICI also remain scarce and many patients develop resistance to ICI, secondarily. The underlying mechanisms at play are not fully understood but involve patient and tumor-intrinsic factors, including tumor microenvironment (TME) components [23, 24]. RCC is a hypervascularized tumor with disorganized vascularization leading to intratumor hypoxia. This induces upregulations of genes involved in metabolism, cell angiogenesis, cell proliferation, and recruitment of immunosuppressive cells as T regulator lymphocytes (T-reg), tumor-associated macrophages (TAM), and myeloid-derived suppressor cells (MDSCs) in the TME [25]. An increased PD-L1 expression in tumor cells is also induced by the release of hypoxia-inducible factor 1-alpha and 1-beta (HIF-1a and HIF-1b) [26]. The vascular endothelial growth factor (VEGF) increases immune checkpoints as CTLA-4, T-cell immunoglobulin and mucin containing protein-3 (TIM3), and lymphocyte-activation gene-3 (LAG3) on T-cells and PD-L1 on dendritic cells, recruits T-reg cells and MDSCs, and suppresses maturation of dendritic cells [24, 27, 28]. All those mechanisms are involved in immunosuppressive processes. Therefore, antiangiogenic treatments are capable of reversing immunosuppression by increasing the tumor-infiltrating lymphocytes and decreasing immunosuppressive cells, immunosuppressive cytokines, and inhibitory molecules on T-cells (PD-1), thus enhancing the efficacy of subsequent or associated ICI therapy [29–31]. Many studies focused on finding reliable biomarkers to enrich ICI responses. While PD-L1 expression and the tumor mutational burden were not associated with better outcomes, compared to melanoma and NSCLC, biomarkers from TME are promising but still need further investigation and validation in RCC [4, 5, 24, 32]. Few clinical data are available regarding TME after a first ICI regimen and subsequent antiangiogenic treatment in the context of ICI rechallenge. However, our results suggest that intervening treatment may decrease PFS at rechallenge. This may be explained by more advanced diseases or by other yet unknown processes in the TME, occurring after a first ICI regimen.RCC is also known to be a heterogenous tumor and may initially present subpopulations of tumor cells with different responsiveness to ICI [22, 23]. Most of our patients received multiples lines of treatments prior to ICI-2, which may have selected subpopulations with weaker immune response, affecting the outcomes of the rechallenge.

The therapy received at rechallenge might also have an impact on the efficacy of ICI rechallenge. As discussed earlier, some studies explored ICIs combination in mRCC and reported higher ORR and PFS. The number of cycles of the combination treatments seems also important. Only two cycles of ipilimumab were administered in the OMNIVORE study, which reported the lowest ORR of 4%, compared to the FRACTION, TITAN, and HCRN GU16-260 studies in which patients received 4 cycles. In our study, monotherapy of nivolumab did not significantly differ from the nivolumab-ipilimumab combination in terms of response rate or PFS at rechallenge, although we lacked power with only three patients rechallenged with this combination.

Rechallenge of ICI was safe in our study with only 4% of serious irAEs during ICI-2, resulting in an 8% risk of recurrence of the initial irAE. In mRCC studies, 13 to 33% of grade ≥3 irAEs have been reported for ICI rechallenge [18–21]. Patients mostly received immune-base combinations in these studies, which may increase toxicity. Larger retrospective studies of Dolladille et al. and Simonaggio et al. reported higher rates of irAEs at rechallenge of 25% and 55%, respectively [10, 11]. However, they included patients with all types of cancer and had a higher rate of immune-base combinations and data collection was not restricted to limiting or grade ≥3 irAEs, which could explain our lower rate. In those studies, only 30 to 40% of patients had a recurrence of their initial irAE. Factors associated with a higher rate of irAE recurrence were the use of anti-CTLA-4 at rechallenge and the type of the irAE (colitis, hepatitis, and pneumonitis), but only few of our patients were concerned by these situations. Moreover, in the present study, only 4 patients were treated with systemic corticosteroids at rechallenge. Two of them started ICI-2 without corticosteroid treatment and developed new irAE (nephritis and adrenal insufficiency), whereas the other two were already under corticotherapy (adrenal insufficiency and analgesic treatment) at start of ICI-2 and did not experience further toxicity. Thus, systemic corticosteroid treatment may improve the safety profile of ICI rechallenge. Of note, selection bias also exists because patients who had experienced a severe or life-threatening irAE (as myocarditis or pneumonitis) might be less prone to receive an ICI rechallenge. Based on our findings, ICI rechallenge seems safe; nevertheless, close monitoring is required for these patients.

Our study has limitations due to its ambispective nature, absence of independent central blinded assessments of the responses and adverse events, and incomplete data collection in some patients. Main limitations of our study are the small sample size and the short follow-up. Although our cohort is national-wide, few patients were eligible to our study. Indeed, rechallenge of ICI is not a common practice, since it is supported by few studies. However, with the higher efficacy of new therapies, clinicians face more often the question of ICI rechallenge in daily practice. Our findings may bring preliminary evidence regarding this strategy. Besides, overall survival may be a better primary endpoint for assessing ICI efficacy; however, we believed that the low number of events related to the small sample size of our population would not allow such analysis. Therefore, we chose ORR as primary endpoint and PFS as criteria for assessing prognostic factors, as they remain practical endpoints and relevant criteria for clinicians and patients at an advanced stage of the disease. Another limitation is the heterogeneity in ICI-1 therapies. Our study was focused on ICI rechallenge with nivolumab and nivolumab-ipilimumab, regardless of the treatment used during ICI-1. In this way, we put our study in the conditions of daily practice for clinicians. We also believed that narrowing our inclusion criteria would decrease the power of the study. Besides, during ICI-1 regimen, patients mostly received nivolumab after front-line therapy which limits the application of our findings, since immune-based combinations are current standard for first-line therapy. Outcomes of a rechallenge may then differ in that setting. Therefore, our findings may fit better to those mRCC patients previously treated with monotherapy of nivolumab after first-line therapy.

## 5. Conclusion

Our study provides real-world data from a nation-wide cohort on rechallenge with nivolumab-based ICI in mRCC patients, showing some efficacy and a good safety profile. We were able to identify various prognostic factors for PFS at rechallenge in univariate analysis but not in multivariate analysis. There were only statistical trends in multivariate analysis for patients with no treatment between ICI regimens and a PFS >6 months at first ICI. These results bring new evidence to this major but still unresolved topic of ICI rechallenge, as well as preliminary, though crucial, data on prognostic factors that may help clinicians in their daily practice. Larger prospective studies are needed to confirm these prognostic factors of response.

## Figures and Tables

**Table 1:** Baseline characteristics of the patients.

Characteristics	*N* = 45, No. (%)
Age at mRCC diagnosis, median (range), yr.	59 (42–90)

*Gender*	
Female	16 (36)
Male	29 (64)

*Smoking status at RCC diagnosis*	
No	24 (59)
Former/active	17 (41)
Missing	4

*Histology*	
Clear cell	41 (91)
Papillary	1 (2)
Other	3 (7)

*Fuhrman or ISUP nuclear grade*	
Low grade (1-2)	8 (21)
High grade (3-4)	30 (79)
Missing	7

*Associated sarcomatoid tumor*	
No	36 (88)
Yes	5 (12)
Missing	4

*Nephrectomy*	
No	6 (13)
Yes	39 (87)

*Timing of nephrectomy*	
Early nephrectomy (at diagnosis)	39 (100)
Local stage	31 (79)
Metastatic stage	8 (21)
Delayed nephrectomy (after systemic treatment)	0 (0)
NA	6

Total number of treatment lines, median (range).	5 (2–10)

mRCC, metastatic renal cell carcinoma; RCC, renal cell carcinoma; ISUP, International Society of Urological Pathology.

**Table 2:** Patients' characteristics at the beginning of ICI-1 and ICI-2 therapies.

Characteristics	ICI-1 (*N* = 45), No. (%)	ICI-2 (*N* = 45), No. (%)
*ECOG performance status*		
0	23 (52)	16 (36)
1	20 (45)	18 (41)
≥2	1 (2)	10 (23)
Missing	1	1

*No. of metastatic sites*		
1	14 (32)	8 (18)
2	15 (34)	12 (27)
≥3	15 (34)	25 (56)
Missing	1	0

*Metastatic sites*		
Lung	31 (74)	32 (76)
Lymph nodes	20 (48)	19 (45)
Liver	10 (24)	15 (36)
Bone	10 (24)	13 (31)
Adrenal gland	4 (10)	9 (21)
Renal	2 (5)	6 (14)
CNS	4 (10)	5 (12)
Other	13 (31)	17 (40)
Missing	3	3

*IMDC score*		
Favorable (0)	12 (29)	9 (23)
Intermediate (1-2)	25 (60)	10 (25)
Poor (3–6)	5 (12)	21 (53)
Missing	3	5

Prior lines of therapy, median (range)	1 (0–6)	3 (1–9)

*Treatment received*		
ICI alone [1]	37 (82)	42 (93)
Nivolumab	35 (78)	42 (93)
Nivolumab-ipilimumab	5 (11)	3 (7)
ICI + TT [2]	3 (7)	0 (0)

Radiotherapy during ICI treatment	5 (11)	5 (11)
Missing	1	0

Systemic corticosteroids use	7 (17)	4 (10)
Missing	4	4

Ongoing ICI	0 (0)	13 (29)
Discontinuation of ICI	45 (100)	32 (71)
Progression	22 (49)	30 (94)
Toxicity	12 (27)	1 (3)
Clinical decision	11 (24)	1 (3)

*Treatment received between ICI regimens*		
0	19 (42)	
1	17 (38)	
≥2	9 (20)	

^1^Two patients received anti-PD-L1 monotherapy: avelumab and atezolizumab. ^2^Treatments received: avelumab + axitinib, nivolumab + tivozanib, and pembrolizumab + lenvatinib. ICI, immune checkpoint inhibitor; ECOG, Eastern Cooperative Oncology Group; CNS, central nervous system; IMDC, International Metastatic Renal Cell Carcinoma Database Consortium; TT, targeted therapy.

**Table 3:** Characteristics of patients who responded to ICI-2.

No.	ICI-1 regimen	IMDC score at ICI-1	Best response at ICI-1	Therapies prior to ICI-1	Reason for ICI-1 discontinuation	ICI-2 regimen	IMDC score at ICI-2	Best response at ICI-2	Therapies between ICI regimens	Treatment-free interval (months)	Ongoing ICI-2 therapy
1	Nivolumab + ipilimumab	Favorable	CR	None	Progression	Nivolumab	Intermediate	CR	Pazopanib	No	Yes
2	Nivolumab + ipilimumab	Poor	PR	None	Toxicity (meningitis)	Nivolumab	Intermediate	PR	No	8.9	Yes
3	Nivolumab	Favorable	PR	Interferon, sunitinib, sorafenib, everolimus,	Clinical decision	Nivolumab	Intermediate	PR	No	12.0	Yes
4	Nivolumab	Intermediate	PR	Pazopanib	Clinical decision	Nivolumab	Intermediate	PR	No	5.0	No (progression)
5	Nivolumab	NA	SD	Sunitinib, pazopanib	Progression	Nivolumab	Favorable	PR	Cabozantinib	No	No (progression)
6	Nivolumab	Intermediate	PD	Everolimus, sunitinib	Progression	Nivolumab	Poor	PR	Sorafenib, nivolumab^2^, cabozantinib	No	No (progression)
7	Nivolumab	Intermediate	PD	Sunitinib, pazopanib, everolimus, sorafenib, bevacizumab, INVAC-1^1^	Progression	Nivolumab	Poor	PR	Sunitinib, cabozantinib	No	No (progression)

^1^INVAC-1: DNA vaccine encoding human telomerase reverse transcriptase (hTERT). ^2^Patient underwent two rechallenges of nivolumab which were independently compared to the first ICI regimen. CR, complete response; PR, partial response; SD, stable disease; PD, progressive disease; IMDC, International Metastatic Renal Cell Carcinoma Database Consortium; ICI, immune checkpoint inhibitor; NA, not available.

## Data Availability

The data are available from the corresponding author upon request.

## Supplementary Materials

Supplementary Materials The following figures and tables can be found in the Supplementary Materials (in order of citation): Table S1: details of clinical decisions that led to ICI-1 discontinuation. Figure S1: flow chart of treatment history of the patients between ICI regimens, regarding the reason for ICI-1 discontinuation. Table S2: efficacy of the first ICI treatment and ICI rechallenge with the response rate and median of DOT, DOR, PFS, and OS. Figure S2: forest plot of the univariate analysis regarding factors associated with PFS at ICI-2. Table S3: report of the immune-related adverse events under ICI-1 and ICI-2.
