# Supplementary material for: REchallenge of NIVOlumab (RENIVO) or Nivolumab-Ipilimumab in Metastatic Renal Cell Carcinoma: An Ambispective Multicenter Study
Source: J Oncol. 2022 Feb 18;2022:3449660. doi: 10.1155/2022/3449660 (PMC8881133; doi:10.1155/2022/3449660)
Supplement: Supplementary Materials — The following figures and tables can be found in the Supplementary Materials (in order of citation): Table S1: details of clinical decisions that led to ICI-1 discontinuation. Figure S1: flow chart of treatment history of the patients between ICI regimens, regarding the reason for ICI-1 discontinuation. Table S2: efficacy of the first ICI treatment and ICI rechallenge with the response rate and median of DOT, DOR, PFS, and OS. Figure S2: forest plot of the univariate analysis regarding factors associated with PFS at ICI-2. Table S3: report of the immune-related adverse events under ICI-1 and ICI-2. [file 3449660.f1.docx]

**SUPPLEMENTARY MATERIALS**

| **Patient no.** | **Best response to ICI-1** | **ICI-1 discontinuation reason** | **Detail** |
| --- | --- | --- | --- |
| 1 | SD | Clinical decision | Non-progressive disease during 8.5 months and patient’s wish. |
| 2 ^1^ | PR | Clinical decision | Non-progressive disease during 4.5 months and patient’s wish. |
| 3 | SD | Clinical decision | Non-progressive disease during 27 months. |
| 4 | CR | Clinical decision | Non-progressive disease during 12 months. |
| 5 | PR | Clinical decision | Non-progressive disease during 18 months and patient’s wish. |
| 6 | PD | Clinical decision | Patient received a treatment course of 6 cycles that was not continued based on his physician’s choice (unknown data). His first assessment scan was 2 months later and showed a progressive disease. |
| 7 | PR | Clinical decision | Patient experienced a partial response under ICI, then underwent surgery, achieving a complete response afterward. ICI was discontinued 20 months after its beginning based on this CR. |
| 8 | CR | Clinical decision | Non-progressive disease during 14 months under ICI. |
| 9 | SD | Clinical decision | NA |
| 10 | PR | Clinical decision | Non-progressive disease during 17 months of ICI. |
| 11 ^2^ | PR | Clinical decision | Non-progressive disease during 19 months of ICI. |

**Table S1.** Details of clinical decisions that led to ICI-1 discontinuation.

*Abbreviations: CR, complete response; PR, partial response; SD, stable disease; PD, progressive disease; ICI, immune checkpoint inhibitor. NA, not available.*

*NOTE: ^1^ Refers to patient no. 3 in Table 3 of main manuscript; ^2^ Refers to patient no. 4 in Table 3 of main manuscript.*

**Figure S1.** Flow chart of treatment history of the patients between ICI regimens.

|  |
| --- |

*Abbreviation: ICI, immune checkpoint inhibitor.*

**Table S2.** Efficacy of the first ICI treatment and ICI rechallenge.

|  | **ICI-1 (N=45)** | **ICI-2  (N=45)** |
| --- | --- | --- |
| **Duration of treatment**, median (95% CI), mo | 5.7 (3.5 - 12.2) | 3.5 (2.5 - 9.8) |
| **Duration of response**, median (95% CI), mo | - | 5.1 (2.7 - NR) |
| **Responses**, no. (%) |  |  |
| Complete response | 4 (9) | 1 (2) |
| Partial response | 19 (42) | 6 (13) |
| Stable disease | 11 (24) | 14 (31) |
| Progressive disease | 11 (24) | 24 (53) |
| **Objective response rate**, % | 51 | 16 |
| **Disease control rate**, % | 76 | 47 |
| **Progression-free survival**, median (95% CI), mo | 11.4 (9.8 - 23.5) | 3.5 (2.8 - 9.7) |
| **Overall survival**, median (95% CI), mo | NR (37.8 - NR) | 24 (9.9 - NR) |

*Abbreviations: NR, not reached; CI, confidence interval; mo, months.*

**Figure S2.** Factors associated to PFS at ICI-2 (univariate analysis).

| **Variables** | **No.** |  |  |  | **HR** | **95% CI** | ***p* value** |
| --- | --- | --- | --- | --- | --- | --- | --- |
| **Gender** |  |  |  |  |  |  |  |
| Male vs. Female | 29/45 |  |  |  | 1.09 | (0.5 - 2.34) | 0.84 |
| **Smoking status** (vs. active) |  |  |  |  |  |  |  |
| Former | 13/41 |  |  |  | 1.79 | (0.47 - 6.81) | 0.39 |
| No | 24/41 |  |  |  | 1.06 | (0.3 - 3.78) | 0.93 |
| **Histological subtype** (vs. clear cell) |  |  |  |  |  |  | 0.61 |
| Papillary | 1/45 |  |  |  | 2.94 | (0.38 - 22.66) |  |
| Other | 3/45 |  |  |  | 0.68 | (0.09 - 5.06) |  |
| **Nuclear grade** (Fuhrman and/or ISUP grade) |  |  |  |  |  |  |  |
| High (grade 3 – 4) vs low (grade 1 – 2) | 38 |  |  |  | 1.17 | (0.43 – 3.16) | 0.76 |
| **Sarcomatoid features** |  |  |  |  |  |  |  |
| Yes vs. no | 5/41 |  |  |  | 1.93 | (0.72 - 5.22) | 0.19 |
| **Nephrectomy** |  |  |  |  |  |  |  |
| Yes vs. no | 39/45 |  |  |  | 0.92 | (0.32 - 2.65) | 0.88 |
| **ECOG-PS at ICI-2** |  |  |  |  |  |  |  |
| 2 - 3 vs. 0 – 1 | 10/44 |  |  |  | 4.81 | (2.05 - 11.26) | < 0.01* |
| **Metastatic sites number at ICI-2** | 45 |  |  |  | 1.45 | (1.1 - 1.9) | < 0.01* |
| **Metastatic sites at ICI-2** (yes vs. no) |  |  |  |  |  |  |  |
| Lung | 32/45 |  |  |  | 1.64 | (0.66 - 4.02) | 0.29 |
| Lymph nodes | 19/45 |  |  |  | 1.38 | (0.66 - 2.89) | 0.40 |
| Liver | 15/45 |  |  |  | 2.55 | (1.17 - 5.58) | 0.02* |
| Bone | 13/45 |  |  |  | 1.48 | (0.7 - 3.14) | 0.31 |
| Adrenal gland | 9/45 |  |  |  | 1.04 | (0.42 - 2.57) | 0.93 |
| Renal | 6/45 |  |  |  | 0.70 | (0.21 - 2.31) | 0.55 |
| Brain | 5/45 |  |  |  | 1.27 | (0.44 - 3.65) | 0.66 |
| Other | 17/45 |  |  |  | 2.01 | (0.95 - 4.27) | 0.07 |
| **IMDC score at ICI-2** (vs. favorable [0]) |  |  |  |  |  |  | < 0.01* |
| Intermediate (1 - 2) | 10/40 |  |  |  | 0.44 | (0.17 - 1.15) |  |
| Poor (3 - 6) | 21/40 |  |  |  | 2.87 | (1.01 - 8.13) |  |
| **Nivolumab monotherapy** (vs. nivolumab-ipilimumab) | 42/45 |  |  |  | 0.53 | (0.12 - 2.36) | 0.41 |
| **Radiotherapy during ICI-2** |  |  |  |  |  |  |  |
| Yes vs. no | 5/45 |  |  |  | 0.43 | (0.13 - 1.44) | 0.17 |
| **Treatment between ICI regimens** |  |  |  |  |  |  |  |
| Yes vs. no | 26/45 |  |  |  | 2.99 | (1.35 - 6.6) | < 0.01* |
| **Reason for ICI-1 discontinuation** (vs. clinical decision) |  |  |  |  |  |  | 0.09 |
| Progression | 22/45 |  |  |  | 2.79 | (1.02 - 7.62) |  |
| Toxicity | 12/45 |  |  |  | 1.87 | (0.62 - 5.65) |  |
| **Blood tests at ICI-2 baseline** |  |  |  |  |  |  |  |
| Hemoglobin | 40 |  |  |  | 1.02 | (0.85 - 1.23) | 0.80 |
| Leukocytes | 38 |  |  |  | 1.19 | (1.01 - 1.4) | 0.04* |
| Neutrophils | 39 |  |  |  | 1.24 | (0.92 - 1.68) | 0.16 |
| Platelets | 38 |  |  |  | 1.01 | (1.0 - 1.01) | < 0.01* |
| Corrected calcium | 35 |  |  |  | 8.19 | (1.28 - 52.38) | 0.03* |
| Albumin | 38 |  |  |  | 0.96 | (0.9 - 1.03) | 0.27 |
| Alkaline Phosphatase (ALP) | 34 |  |  |  | 1.00 | (1.0 - 1.01) | 0.45 |
| Lactate Dehydrogenase (LDH) | 32 |  |  |  | 1.01 | (1.0 - 1.01) | < 0.01* |
| C-Reactive Protein (CRP) | 28 |  |  |  | 1.01 | (1.0 - 1.02) | 0.08 |
| **ICI-1 PFS** (vs. ≤ 6 months) |  |  |  |  |  |  | < 0.01* |
| 6 - 12 months | 7/45 |  |  |  | 0.40 | (0.15 - 1.01) |  |
| > 12 months | 15/45 |  |  |  | 0.16 | (0.07 - 0.4) |  |
|  |  |  |  |  |  |  |  |
|  |  |  |  |  |  |  |  |

*Abbreviations: HR, hazard ratio; CI, confidence interval; ISUP, International Society of Urological Pathology; ECOG, Eastern Cooperative Oncology Group; PS, performance status; IMDC, International Metastatic Renal Cell Carcinoma Database Consortium; ICI, immune checkpoint inhibitor.*

**Table S3.** Immune-related adverse events under ICI-1 and ICI-2.

|  | **ICI-1**  **(N=45)** | | | **ICI-2**  **(N=45)** | | |
| --- | --- | --- | --- | --- | --- | --- |
| **Event ^1^** | Any grade no. (%) | Grade 3 no. (%) | Grade 4 no. (%) | Any grade no. (%) | Grade 3 no. (%) | Grade 4 no. (%) |
| All events | 13 (29) | 9 (20) | 2 (4) | 2 (4) | 2 (4) | 2 (4) |
| Hypophysitis | 2 (4) | 0 (0) | 1 (2) | 0 (0) | 0 (0) | 0 (0) |
| Hepatitis | 1 (2) | 0 (0) | 1 (2) | 0 (0) | 0 (0) | 0 (0) |
| Pneumonitis | 2 (4) | 2 (4) | 0 (0) | 0 (0) | 0 (0) | 0 (0) |
| Lichen planus | 2 (4) | 2 (4) | 0 (0) | 0 (0) | 0 (0) | 0 (0) |
| Nephritis | 2 (4) | 1 (2) | 0 (0) | 1 (2) | 1 (2) | 0 (0) |
| Psoriasis | 1 (2) | 1 (2) | 0 (0) | 1 (2) | 1 (2) | 0 (0) |
| Eosinophilia | 1 (2) | 1 (2) | 0 (0) | 0 (0) | 0 (0) | 0 (0) |
| Hypopituitarism | 1 (2) | 1 (2) | 0 (0) | 0 (0) | 0 (0) | 0 (0) |
| Meningitis | 1 (2) | 1 (2) | 0 (0) | 0 (0) | 0 (0) | 0 (0) |

NOTE: ^1^ Type and grade of events according to the Common Terminology Criteria for Adverse Events version 5.0.
